# Enhancing growth performance in Liangshan black sheep through fermented onion: insights from transcriptomics and metabolomics

**DOI:** 10.3389/fvets.2025.1533728

**Published:** 2025-05-15

**Authors:** Chaoyun Yang, Shuzhe Wang, Yunxia Qi, Yadong Jin, Ran Guan, Zengwen Huang

**Affiliations:** College of Animal Science and Technology, Xichang University, Xichang, China

**Keywords:** fermented onion, Liangshan black sheep, transcriptome, metabolomics, rumen, average daily gain

## Abstract

The objective of this study was to assess the effect of fermented onion (FO) on the growth performance of Liangshan black sheep (LBS) and to elucidate its potential molecular mechanisms from a multi-omics perspective. A total of 20 LBS were randomly assigned to one of four groups and fed diets containing 0, 10, 20%, or 30% fermented onions, respectively. The initial and final body weights were recorded. Following the termination of the experiment, the control group and the group exhibiting the most significant increase in average daily gain (ADG) were selected for slaughter. Rumen epithelial tissue was then collected for transcriptome sequencing, while fermented and unfermented onions were collected for untargeted metabolomics. The study revealed that the supplementation of 20% FO led to a notable enhancement in the ADG of LBS, whereas the addition of 30% resulted in a growth-inhibitory effect. Metabolomic analysis revealed that the fermentation process markedly elevated the concentration of bioactive compounds in the onion, including quercetin, rutin, luteolin, myricetin, 4′-methoxyflavone and other flavonoids, as well as linoleic acid, *γ*-linolenic acid and diverse amino acids. Transcriptome analysis revealed 34 differentially expressed genes (DEGs), which were primarily enriched in protein-related signaling pathways, glycerolipid metabolism, and digestion and absorption-related pathways. The appropriate addition of FO has been demonstrated to promote the growth performance of LBS by increasing the concentration of bioactive substances and regulating metabolic processes and gene expression. The findings of this study provide a scientific basis for improving the growth performance of LBS and making more effective use of onion resources, and contribute new insights to the development and utilization of feeds.

## 1 Introduction

The Sichuan Liangshan black sheep (LBS) is a distinctive local sheep breed originating from the Liangshan Yi Autonomous Prefecture in Sichuan Province, China. Renowned for its exceptional adaptability to high-altitude, low-oxygen environments, this breed is also celebrated for producing premium-quality meat with high nutritional value and resilience to harsh feed conditions (1). The LBS holds significant cultural importance for the Yi people, who constitute approximately 48.8% of the region’s total population (2). It plays a vital role in traditional rituals, such as sacrifices to honor ancestors and weddings as a symbol of good fortune. Biologically, the LBS exhibits remarkable resilience and disease resistance, enabling it to thrive and reproduce in highland environments at altitudes ranging from 2,000 to 3,500 m (3, 4). Its meat is distinguished by its tenderness, juiciness, and unique flavor, along with a high content of essential amino acids and unsaturated fatty acids. Despite these advantages, the breed faces certain challenges, particularly its relatively slow growth rate and a high incidence of single lambs, which limit its production efficiency. Therefore, enhancing the growth rate of the LBS has become a critical goal for improving its productivity.

The challenge of improving LBS growth performance is further complicated by the broader issue of food security. With the global population steadily increasing, the competition between humans and animals for food resources has intensified. The development and utilization of agricultural by-products have emerged as an effective strategy to alleviate this problem. According to statistics from the Food and Agriculture Organization of the United Nations, approximately one-third of the global food supply—around 1.3 billion tons—is wasted during production and consumption phases (5, 6). Efficiently utilizing these by-products can not only mitigate the pressure on food supplies but also reduce environmental pollution and promote resource recycling.

Liangshan Prefecture, known as the “hometown of Chinese onions,” produces a substantial quantity of onions and related by-products each year. Onions are rich in bioactive compounds such as quercetin (7, 8), allicin, and polyphenolic compounds (9, 10). These substances have demonstrated various beneficial properties, including anti-inflammatory, antioxidant, and digestion-enhancing effects, as well as the ability to improve animal growth performance (11–16). However, onions also contain pungent sulfur compounds, such as dipropyl disulfide (17), allyl sulfides (17), thiosulfinates, and pyruvates (18). These compounds can negatively impact livestock feed intake and consequently hinder growth performance due to their sharp, spicy flavor.

To address these challenges, researchers have begun exploring various processing techniques, such as fermentation and heat treatment, to reduce the pungency and toxicity of onions while preserving their beneficial bioactive components (19, 20). These methods have shown potential in improving the palatability, nutritional value, and bioactive substance content of onions, making them more suitable for animal feed. The objective of this study was to evaluate the effects of fermented onion (FO) on the growth performance of LBS and to elucidate the potential molecular mechanisms underlying these effects from a multi-omics perspective. This research aims to provide a scientific basis for improving LBS production efficiency and promoting the efficient utilization of onion resources.

## 2 Materials and methods

### 2.1 Preparation of fermented onion

The onion should be peeled, the head and roots removed, and the remaining portion chopped into pieces of approximately 1.5 cm × 1.5 cm in size. Subsequently, 5% sugar and 1% salt should be added, and the mixture should be agitated until the onion juice is released. Once the mixture has been thoroughly combined, it should be transferred to a food-grade fermentation container and placed in a cool environment. During the summer months, the container should be left at room temperature for a period of 25 days, during which time the fermentation process will occur in an anaerobic environment. A successfully fermented onion (FO) will manifest the following characteristics: it will be soft in texture, exude a sour, robust aroma, and display no pungent odor or evidence of mildew.

### 2.2 Animal husbandry management

The experiment applied a single-factor test design. Twenty four-month-old female LBS (weighing 23 ± 0.5 kg) in good health were selected and randomly divided into 4 groups, with 5 subjects in each. The control group (Con) was fed a diet that was 100% fully complete (Xinjiang Taikun Group, China), while the experimental groups were fed a diet that was supplemented with 10% (Trt1), 20% (Trt2), and 30% (Trt3) FO, respectively. The sheep were provided with ad libitum access to food and water. A pre-test period of 15 days was followed by a formal trial period of 60 days. The animals were fed at 11:00 a.m. and 16:00 p.m. each day. After the experiment, calculate the average daily gain (ADG) using the formula ADG = (BWt – BW0)/d, where BWt is the final body weight (kg), BW0 is the initial body weight (kg), and d is the number of experimental days.

### 2.3 Tissues collection

Fermented onion and fermentation liquid from each of the six barrels were extracted and placed in cryogenic tubes, numbered and immediately placed in liquid nitrogen for untargeted metabolomic detection. The procedures were conducted in accordance with the guidelines set forth by the Animal Care Committee of Xichang University (No. XCU20230925). At the conclusion of the experiment, the group exhibiting the most significant alteration in average daily gain (ADG) and the control group were selected for slaughter, and rumen tissue was collected. The rumen tissue was washed with PBS, chopped, and then placed in numbered 2 mL cryogenic tubes and placed in liquid nitrogen for total RNA extraction for transcriptome sequencing.

### 2.4 Transcriptome sequencing and bioinformatics analysis

#### 2.4.1 RNA isolation, cDNA library construction and transcriptome sequencing

Following the completion of the ADG calculation, the 10 animals from the 20% FO and 0% FO group were slaughtered after a 16-h fasting period, in compliance with the guidelines established by the Animal Ethics Committee at Xichang University. Immediately following slaughter, rumen tissue samples were procured and rinsed with PBS solution. It was then divided into smaller pieces and placed in sterile tubes that had been treated to prevent the contamination of nucleic acids, and subsequently stored in liquid nitrogen. In concordance with the protocol outlined in the TRIzol RNA extraction kit (Invitrogen, Carlsbad, CA, United States), around 500 mg of rumen tissue was procured for RNA extraction. The quality of the RNA extracted from the 10 samples was found to be satisfactory, with a RNA quality score > 1.8 and an RIN value > 7. cDNA libraries were constructed and sequenced by Chenqi Biotechnology Co., Ltd. (Shanghai, China) employing the Illumina HiSeq 4,000 platform (Illumina, San Diego, California, United States) with a sequencing length of 150 bp and a pair-end configuration.

#### 2.4.2 Quality control and alignment

The assessment of sequencing quality was undertaken utilizing the FastQC software (version 0.11.7^1^). Consequently, reads containing standard adaptors or poly-N sequences, in addition to low-quality reads, were subjected to trimming through the application of the Trim-galore software (version 0.6.6^2^). Subsequently, reads with an QC > 20 were selected for further analysis. Construction of the bovine genome index was undertaken with the HISAT2 software (version 2.2.1^3^). Subsequently, the clean reads were aligned with the reference genome^4^. The sheep reference genome and annotation files, obtained from the UCSC database, were employed to assist in the quantification of gene using the StringTie software (version 2.1.2^5^). Finally, gene expression was quantified using FPKM value.

#### 2.4.3 Identification and functional annotation of differentially expressed genes (DEGs)

A principal component analysis (PCA) was subsequently implemented to evaluate the reproducibility of the samples following the determination of their FPKM value. Consequently, six owes, comprising three individuals from each group, were subjected to differential expression analysis. The identification of differentially expressed genes (DEGs) was accomplished through the implementation of the R package DESeq2 (version 1.24.0^6^). The fold change (FC) thresholds for identifying DEGs were set at |Log2FC| ≥ 1 and the false discovery rate (FDR) < 0.05.

The gene function annotation and visualization were conducted using the gene ontology (GO) analysis function within the R package clusterProfiler (version 4.05^7^). Gene Ontology annotation, encompassing three domains: biological process (BP), molecular function (MF), and cellular component (CC), was undertaken through the enrichGO function. The enrichKEGG function was applied to annotate the potential signaling pathways in which the DEGs might be involved, leveraging the Kyoto Encyclopedia of Genes and Genomes (KEGG). The R package ggplot2 was harnessed to visualize the entirety of the results generated by the enrichment analysis. The statistical significance of each GO term and KEGG pathway was ascertained using the Fisher test, with a threshold for *p*-values < 0.05.

#### 2.4.4 Protein–protein interaction (PPI) network analysis

Subsequently, the DEGs were mapped to the STRING database (version 11.0^8^) for the purpose of retrieving information regarding their interactions. A confidence score >0.9 was deemed sufficient to select the relevant modules. The Cytoscape software (version 3.6.1^9^) was used to visualize and construct complex interaction networks. The CytoHubba plugin in Cytoscape was used to determine hub genes through four centrality methods: network topology analysis (Degree), edge percolated component (EPC), maximal clique centrality (MCC), and maximum neighborhood component (MNC) (21). Subsequently, the genes identified by all four methods were designated as the core gene set. The MCODE plugin was then used to determine pivotal sub-networks and the nodes. These were subsequently identified as the hub gene set (degree cutoff = 2, node score cutoff = 0.2, k-core = 2, and maximum depth = 100). Finally, the genes from each module were subjected to GO and KEGG enrichment analyses.

### 2.5 Metabolomics and bioinformatics analysis

#### 2.5.1 UHPLC-Q-Exactive Orbitrap MS

The analysis was conducted using an ultra-high-performance liquid chromatography (UHPLC) system (Vanquish UHPLC, Thermo) coupled to an Orbitrap mass spectrometer (Q Exactive HF-X/Q Exactive HF) at CHI BIOTECH CO., LTD. Hydrophilic interaction liquid chromatography (HILIC) was conducted using a 2.1 mm × 100 mm ACQUITY UPLC BEH Amide 1.7 μm column (Waters, Ireland).

The mobile phase for both positive and negative electrospray ionization (ESI) modes consisted of two solvents: solvent A (25 mM ammonium acetate and 25 mM ammonium hydroxide in water) and solvent B (acetonitrile). The gradient elution profile was as follows: the initial conditions of 98% B were maintained for 1.5 min, followed by a linear decrease to 2% B over 10.5 min. Subsequently, a two-minute isocratic period was conducted at 2% B. Thereafter, the gradient was returned to 98% B within 0.1 min, followed by a three-minute re-equilibration period.

The ESI source parameters were optimized as follows: The parameters for the gas1 and gas2 were set at 60, the curtain gas (CUR) at 30, the source temperature at 600°C, and the IonSpray Voltage Floating (ISVF) at ±5,500 V. For MS acquisition, the instrument was configured to collect data over an m/z range of 80–1,200 Da, with a resolution of 60,000 and an accumulation time of 100 ms. In auto MS/MS mode, the instrument acquired data over an m/z range of 70–1,200 Da, with a resolution of 30,000 and an accumulation time of 50 ms. An exclusion time of 4 s was implemented to enhance the coverage of unique precursor ions.

#### 2.5.2 Data processing

The unprocessed MS data were transformed into MzXML format using the ProteoWizard MSConvert tool prior to importation into the open-source XCMS software (version 3.20^10^). Peak picking was conducted using the following parameters: centWave m/z = 10 ppm, peakwidth = c (10, 60), and prefilter = c (10, 100). The parameters for peak grouping were set to bw = 5, mzwid = 0.025, and minfrac = 0.5.

Isotope and adduct annotation were performed using the CAMERA (version 3.20^11^) software. Only variables exhibiting a minimum of 50% non-zero measurement values in at least one group were retained for further analysis in the extracted ion features. Metabolite identification was conducted by comparing the accuracy of m/z values and MS/MS spectra with an in-house database constructed using available authentic standards.

#### 2.5.3 Statistical analysis

Following sum-normalization, the processed data were subjected to multivariate data analysis using the R package (ropls^12^). This included Pareto-scaled principal component analysis (PCA) and orthogonal partial least-squares discriminant analysis (OPLS-DA). The robustness of the model was evaluated through the application of a 7-fold cross-validation and response permutation testing. The variable importance in the projection (VIP) value of each variable in the OPLS-DA model was calculated in order to indicate its contribution to the classification. A Student’s *t*-test was employed to ascertain the statistical significance of the observed differences between the two groups of independent samples. Metabolites exhibiting a VIP ≥ 1 and a *p*-value < 0.05 were identified as differentially expressed metabolites (DEMs). Pearson’s correlation analysis was conducted to assess the relationship between two variables.

#### 2.5.4 Bioinformatic analysis of DEMs

In order to provide a more comprehensive and intuitive representation of the relationships between samples and the differences in the expression patterns of metabolites in different samples, a distance matrix was calculated for the expression levels of all samples and DEMs. Subsequently, a cluster analysis was conducted using the hierarchical clustering method. The resulting clustering is presented in the form of a heat map, which facilitates straightforward observation. Subsequently, a correlation analysis was conducted to ascertain the regulatory relationships between metabolites.

In order to elucidate the systematic effects and potential mechanisms of DEMs, the DEMs were mapped to the KEGG database with the objective of identifying the signal pathways in which they were involved. The process offers potential targets for further research or therapeutic intervention. Finally, the R language package ggplot2 (version 3.3.2^13^) was employed to enhance the visual presentation of the signal pathways, thereby providing a more comprehensive and readily comprehensible representation of the results.

### 2.6 Validation of RNA-seq via quantitative real-time PCR (qPCR)

To validate RNA-seq results, four differentially expressed genes (*MyHC*, *MS4A1*, *CAT*, and *MyoG*-2) were randomly selected for qPCR verification. Primers were designed using primer-blast tool and synthesized by Sangon (China). First-strand cDNA was synthesized from 1 μg total RNA of each sample using One-Step gDNA Removal and cDNA Synthesis SuperMix (Vazyme, China). qPCR was performed using CFX Real-Time PCR system with SYBR Green Master Mix. Each sample was tested in triplicate. *GAPDH* served as internal reference, and relative expression was calculated using the 2^-ΔΔCt^ method. Primer sequences and detailed qPCR protocols are available in Supplementary Tables S1, S2.

## 3 Results

### 3.1 Comparative analysis of FO supplementation on LBS growth metrics

**Figure 1:**
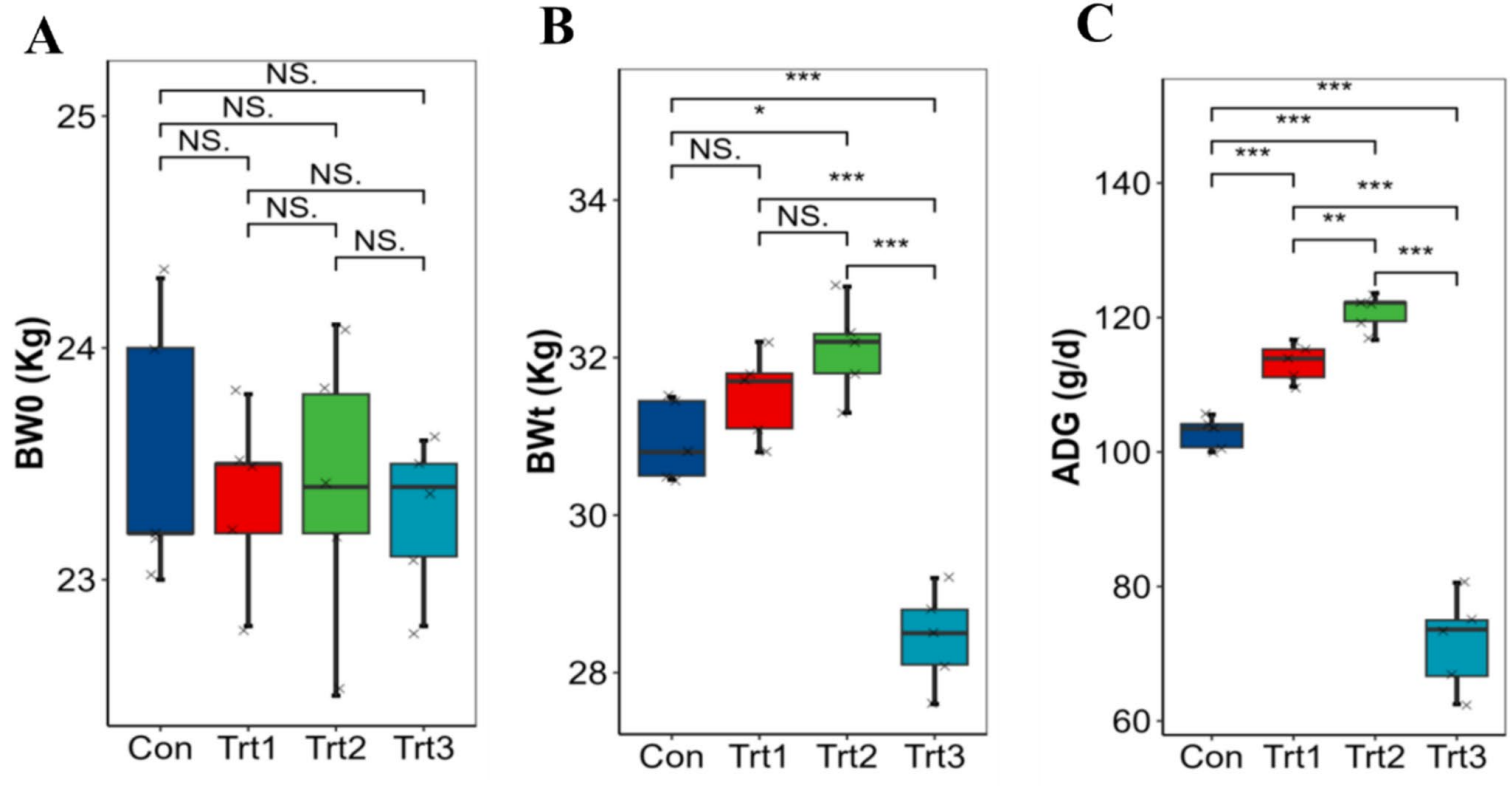
Effect of FO on the ADG of LBS **(A)** BW0 = initial body weight; **(B)** BWt = teminal body weight; **(C)** ADG = avaerage daily gain. *p <0.05; **p <0.01; ***p <0.001.

The growth performance of LBS was affected by the feeding of different proportions of fermented onions, provided that there was no significant difference in initial BW0 (Figure 1A). The results demonstrated that, in comparison to the Con group, the BWt of the Trt2 group was significantly higher than that of the Con group and the Trt3 group (Figure 1B). Conversely, the growth performance of the Trt3 group was significantly inhibited, and its BWt was markedly lower than that of the remainder of the groups (*p* < 0.01). The analysis of the difference in ADG (Figure 1C) revealed that the Trt2 group exhibited an extremely significant increase in growth compared to the other three groups (*p* < 0.01), with an average of 120.8 g/d. The Trt1 group demonstrated an extremely significant increase in growth compared to the Con and Trt3 groups (*p* < 0.01). The results demonstrate that the addition of FO at a moderate level can have a positive effect on the growth of LBS. However, excessive amounts of FO have been observed to have a suppressive and detrimental impact on the growth performance of the LBS.

### 3.2 Metabolomic profiling of FO: alterations in bioactive substance concentrations

**Figure 2:**
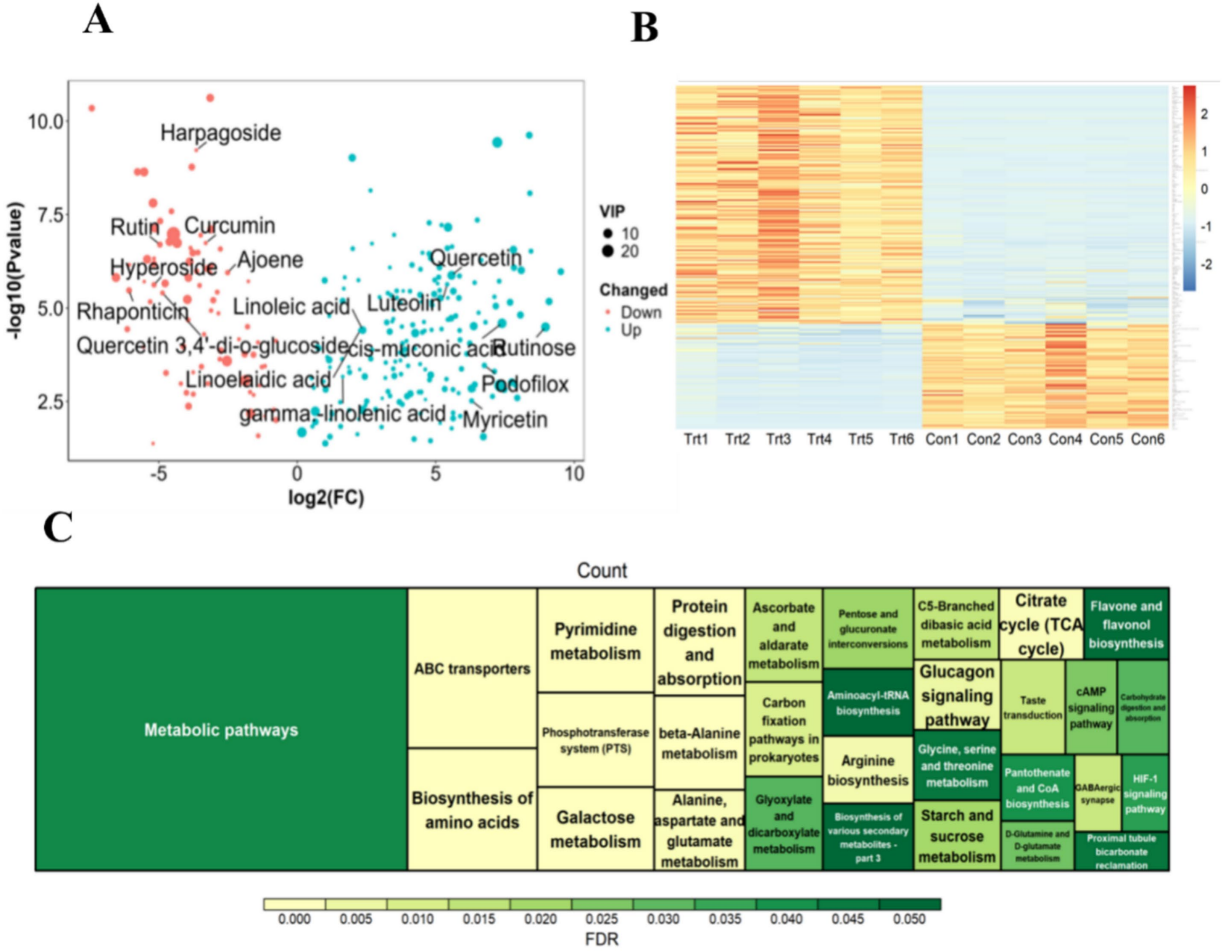
Metabolite difference analysis and its enrichment analysis. **(A)** Volcano plot of differential metabolites. **(B)** Heatmap of differential metabolites. **(C)** Functional enrichment analysis of differential metabolites.

A metabolomic analysis of fermented onions was implemented to ascertain alterations in the concentration of bioactive substances present in the onions during the fermentation process. A total of 11,228 metabolites were identified (Supplementary Table S3). In comparison with the Con group (Figure 2A and Supplementary Table S3), a total of 165 differential metabolites were identified in the cationic state (screening conditions VIP ≥ 1, FC ≥ 2, *p*-value < 0.05). In the anionic state, a total of 90 differential metabolites were identified, including 58 up-regulated and 32 down-regulated metabolites. Among the differential metabolites, flavonoid compounds such as quercetin, rutin, kaempferol, and luteolin exhibited a notable upregulation (Figures 2A,B). Additionally, the downregulated differential metabolites, including saponins, hyperosides, rutin, and quercetin 3,4′-di-o-glucoside, demonstrated a substantial downregulation (Figures 2A,B).

An enrichment analysis was conducted on the significantly differentially expressed metabolites to provide insight into their potential functions. The results demonstrated that 255 differentially expressed metabolites were significantly enriched in 49 signal pathways, with the metabolic pathways exhibiting the highest degree of enrichment (Figure 2C). Further analysis revealed that the differential metabolites were predominantly enriched in amino acid metabolism-related signaling pathways, including ‘d-Glutamine and d-glutamate metabolism’, ‘Aminoacyl-tRNA biosynthesis’, ‘Alanine, aspartate and glutamate metabolism’, and so on. This finding is consistent with the established fact that amino acids are the fundamental building components of proteins and are involved in a multitude of biological processes. The differential metabolites are largely the result of amino acids and their condensation products (Supplementary Table S3). Additionally, the signal pathways related to sugar metabolism, including ‘Fructose and mannose metabolism’, ‘Pyruvate metabolism’, ‘TCA cycle’, ‘Insulin secretion’, and ‘Glucagon signaling pathway’, are of interest. It is noteworthy that this study also enriched the flavonoid metabolic pathway ‘Flavone and flavonol biosynthesis’, which is consistent with the results of multiple flavonoid differential metabolites mentioned above. Additionally, the ‘HIF-1 signaling pathway’ and ‘cAMP signaling pathway’ and other signal pathways were also enriched. The aforementioned results demonstrate that anaerobic fermentation of onions can markedly affect the concentration of amino acids and flavonoid active substances in onions, which may be a contributing factor to the observed increase in ADG of LBS.

### 3.3 The expression and functional changes of LBS rumen epithelial genes are modified by FO

**Figure 3:**
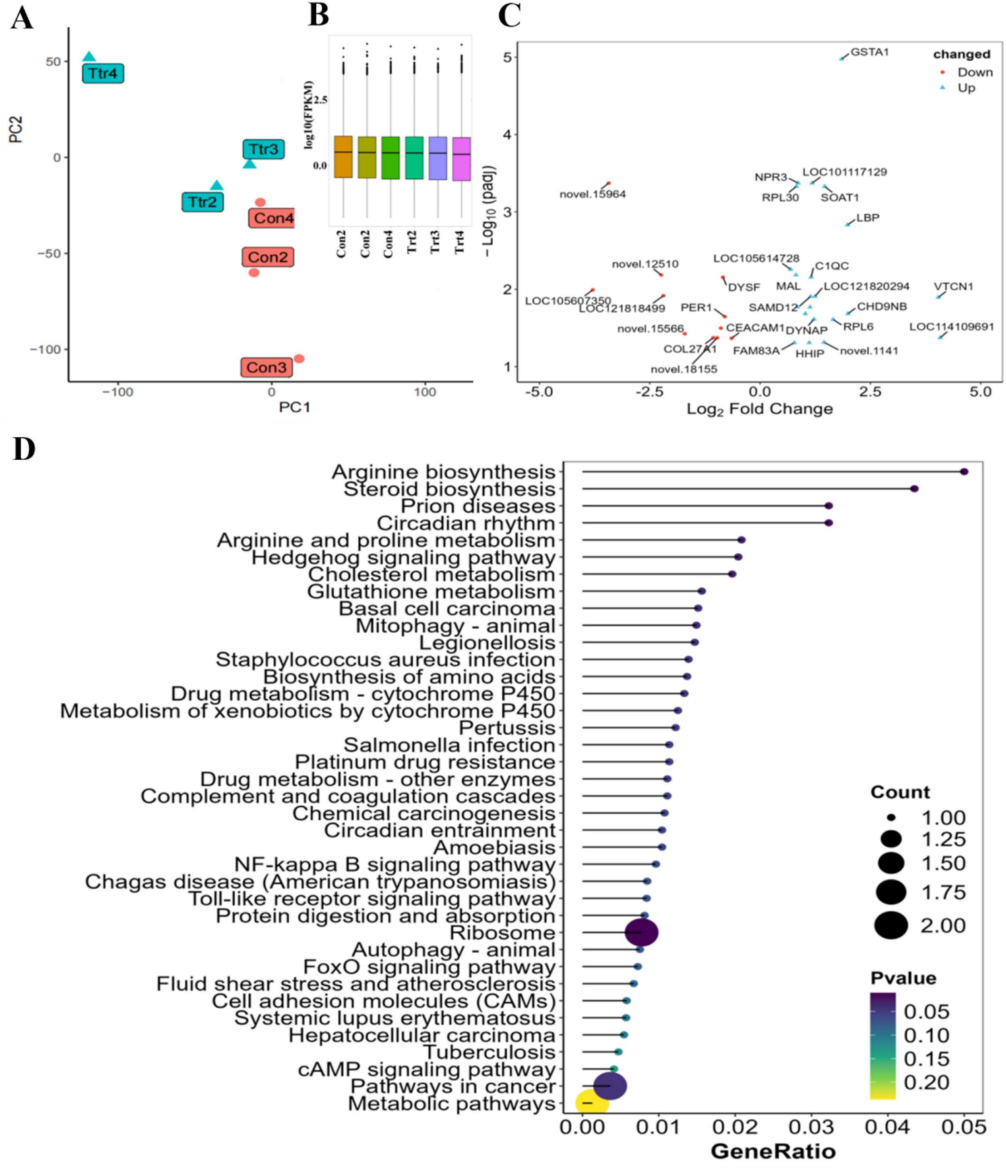
differential expressed analysis of gene and its enrichment analysis. **(A)** Sample grouping PCA plot; **(B)** sample sequencing surge plot; **(C)** differential gene Volcano plot; **(D)** differential gene matchstick plot.

The number of clean reads pairs obtained through sequencing (Supplementary Table S4) ranged from 21,110,544 to 31,084,542. The false discovery rate of clean reads was less than FDR (Q30) < 0.001, and the GC content variation range was 50.26–52.03%. The principal component analysis (Figure 3A) demonstrated that there were notable discrepancies between the control and experimental groups, and that the intra-group reproducibility was satisfactory. The results of the differential analysis (Figure 3B) demonstrated that a total of 34 differentially expressed genes (DEGs) were identified (Figure 3C), comprising 22 up-regulated genes and 12 down-regulated genes.

A further analysis of the functions of these DEGs revealed that they were mainly enriched in protein-related signaling pathways (Figure 3D), including ‘Biosynthesis of amino acids’, ‘Protein digestion and absorption’, ‘Arginine and proline metabolism’, ‘Arginine biosynthesis’, and ‘Ribosome’. Significantly, the differential analysis revealed the involvement of glycolipid metabolism-related signal pathways, such as ‘Glutathione metabolism’, ‘Cholesterol metabolism’, and ‘Steroid biosynthesis’, as well as digestion and absorption-related signal pathways, such as ‘cAMP signaling pathway’, ‘FoxO signaling pathway’, ‘Toll-like receptor signaling pathway’, and ‘NF-kappa B signaling pathway’. Additionally, other signal pathways were identified, including drug metabolism and bacterial metabolism. These included pathways related to drug metabolism by cytochrome P450, other enzymes, platinum drug resistance, and circadian rhythm.

### 3.4 Validation of RNA-seq results

**Figure 4:**
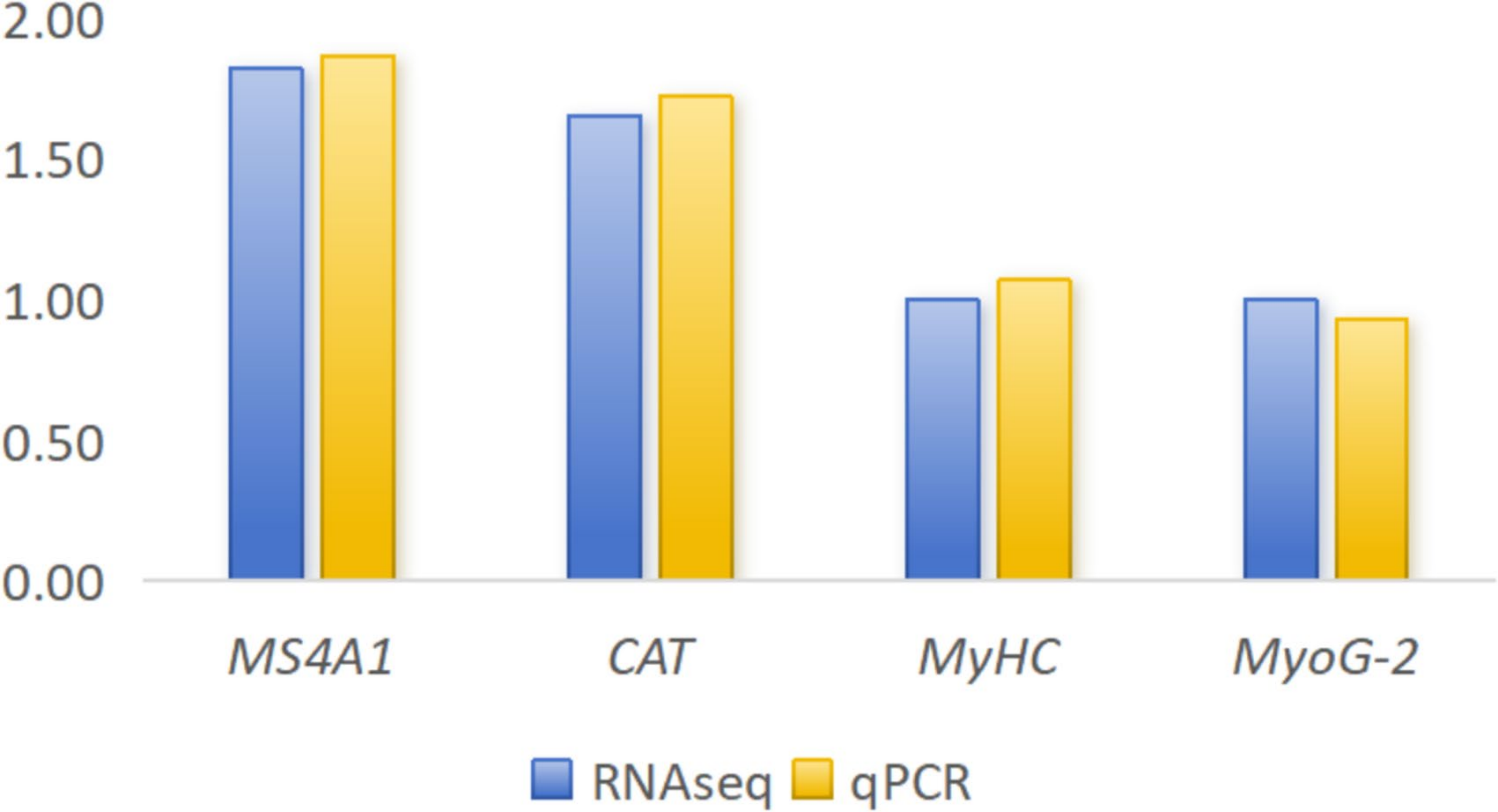
Quantitative real-time PCR (qPCR) results for four randomly selected genes.

To validate the accuracy of RNA-seq data, we randomly selected four genes for validation (*MyHC*, *MS4A1*, *CAT*, and *MyoG*-2). The qPCR analysis was performed using the same RNA samples that were used for RNA-seq. Results demonstrated that expression patterns of these genes were consistent between qPCR and RNA-seq methods, confirming the reliability and accuracy of our transcriptome data (Figure 4).

## 4 Discussion

The utilization of inexpensive and discarded agricultural products or by-products as livestock feed represents a significant strategy for the alleviation of food shortages. Effective processing methods, particularly fermentation, play a crucial role in enhancing the utilization efficiency of these by-products. Fermentation has been reported to improve the nutritional profile of plant-based materials by increasing the bioavailability of amino acids, flavonoids, and other bioactive compounds, thereby enhancing their biological activity (22).

The objective of this study was to elucidate the molecular mechanism by which fermented onions (FO) promote the average daily gain (ADG) of Liangshan black sheep (LBS). Our findings demonstrate that the addition of 20% FO resulted in a notable increase in the ADG of LBS. Following the fermentation process, the biologically active compounds present in onions, including quercetin, luteolin, rutinose, myricetin, podofilox, linoleic acid, *γ*-linolenic acid, and various amino acids, exhibited a significant increase in their concentrations. Previous studies have indicated that fermentation can enhance the antioxidant properties and biological activity of onions by increasing the concentration of flavonoids and polyphenols (23, 24). Furthermore, the fermentation process has been shown to augment the amino acid profile of onions, particularly those associated with the glutamic acid, aspartic acid, and arginine families, as demonstrated by Tan et al. (22).

The enhancement of amino acid concentration may be attributed to the hydrolysis of proteins during fermentation, improving the nutritional value of FO. Additionally, our study revealed that the arginine and lysine biosynthesis pathways were activated, and the *ARG1* gene was significantly upregulated in rumen epithelial cells following FO feeding. This finding indicates that the fermentation process not only increases the amino acid content but also influences gene expression in rumen epithelial cells. These results align with findings from previous research where fermentation was reported to modulate gene expression related to metabolic and immune functions in animals (25, 26).

Onions are known to be a rich source of bioactive substances, and their fermentation can further enhance these properties. For example, Yang et al. (23) demonstrated that fermentation increased the antioxidant capacity of onions by boosting their polyphenol and flavonoid content (24). The current study confirmed these findings, with a significant increase in the concentrations of quercetin, luteolin, rutinose, myricetin, and other compounds. Moreover, the production of short-chain fatty acids (SCFAs) such as acetic, propionic, and butyric acids during fermentation has been reported to benefit intestinal health and promote animal growth (24).

The quercetin content of FO increased by a factor of 47.6. As a naturally occurring flavonoid, quercetin has been shown to improve growth performance and health in various animal species. For instance, quercetin supplementation in poultry has been reported to enhance growth performance, immune function, and antioxidant capacity (27–30). Additionally, quercetin has been found to alleviate heat stress in broilers and promote gut health by enhancing the growth of beneficial bacteria like Lactobacillus (30, 31). However, its effects are dose-dependent, with higher levels potentially leading to adverse outcomes (32). The results of this study align with previous findings, where 20% FO enhanced LBS growth, while 30% FO resulted in growth inhibition.

Furthermore, fermentation significantly increased the levels of linoleic acid and *γ*-linolenic acid in FO. These fatty acids are known to have various health benefits, including anti-inflammatory and antioxidant effects. Studies have shown that increasing dietary linolenic acid can enhance egg production and improve immune function in animals (33–35). Linoleic acid, on the other hand, has been reported to improve feed efficiency and promote growth in livestock when administered appropriately (36, 37).

The increased arginine concentration and significant upregulation of the *ARG1* gene observed in this study are noteworthy. Arginine is an essential amino acid involved in various physiological functions, including protein synthesis, nitric oxide production, and immune function. Dietary arginine supplementation has been shown to enhance growth performance, antioxidant capacity, and gut health in poultry and other animals (38–40). Moreover, arginine is critical for muscle development, energy metabolism, and overall immune function (41–43). The upregulation of the *ARG1* gene may indicate enhanced arginine metabolism, contributing to the improved growth performance of LBS fed with FO. Our findings also suggest that FO supplementation modulates the rumen microbiota and metabolic pathways. Previous studies have reported that fermented feed can enhance nutrient digestibility, improve gut health, and promote growth performance by modulating gut microbiota (25, 26, 44–48). Additionally, the synergistic effects of bioactive compounds and SCFAs produced during fermentation may further enhance animal growth.

In conclusion, our study demonstrates that FO supplementation at an optimal level (20%) can significantly enhance the growth performance of LBS. This improvement is likely due to the increased concentration of bioactive compounds, such as quercetin, and their influence on gene expression related to metabolic processes. However, excessive FO supplementation may have adverse effects, as evidenced by the reduced growth performance observed in the 30% FO group. Further research should focus on long-term feeding trials, safety assessments, and the impact of FO on meat quality to fully explore its potential in animal husbandry.

## 5 Conclusion

The findings of this study indicate that the incorporation of FO at an optimal level can markedly enhance the growth performance of LBS. The data suggests that an addition of 20% is the most efficacious. Metabolomic and transcriptomic analyses demonstrated an increase in the concentration of bioactive compounds in FO and their impact on the gene expression of LBS rumen epithelial cells, offering novel insights for enhancing the production efficiency of LBS and leveraging agricultural by-products. Nevertheless, this research is not without limitations, including the absence of long-term feeding trials and an in-depth investigation of the impact on meat quality. Further research should concentrate on the long-term effects and safety of FO, investigate the mechanisms of action of key active substances in greater depth, examine the potential synergies with other feed additives, and expand the scope of research to other livestock and poultry species in order to fully realize the potential of FO in animal husbandry.

## Data Availability

Raw data was uploaded to SRA database,the accession was PRJNA1192505.

## Footnotes

^1^ https://www.bioinformatics.babraham.ac.uk/projects/fastqc/

^2^ https://www.bioinformatics.babraham.ac.uk/projects/trim_galore/

^3^ http://daehwankimlab.github.io/hisat2/

^4^ https://www.ncbi.nlm.nih.gov/datasets/genome/GCF_000298735.2/

^5^ http://ccb.jhu.edu/software/stringtie/

^6^ http://www.bioconductor.org/packages/release/bioc/html/DESeq2.html

^7^ https://www.bioconductor.org/packages/release/bioc/html/clusterProfiler.html

^8^ https://string-db.org/

^9^ https://cytoscape.org/

^10^ https://www.bioconductor.org/packages/release/bioc/html/xcms.html

^11^ https://www.bioconductor.org/packages/release/bioc/html/CAMERA.html

^12^ https://www.bioconductor.org/packages/release/bioc/html/ropls.html

^13^ https://ggplot2.tidyverse.org/
